# 4′-Chloro-3′,5′-dimethoxy­acetanilide

**DOI:** 10.1107/S1600536809013166

**Published:** 2009-04-22

**Authors:** Huiyu Li, Yanyan Zhu, Zhonghua Chen

**Affiliations:** a Department of Mathematics and Physics, Shanghai University of Electric Power, Shanghai 201300, People’s Republic of China

## Abstract

The title compound, C_10_H_12_ClNO_3_, crystallizes with four independent mol­ecules in the asymmetric unit which are linked by inter­molecular N—H⋯O hydrogen bonds.

## Related literature

The natural pyran­oacridone acronycine, which can be synthesized from the title compound, exhibits a broad spectrum of activity against numerous experimental tumor models, see: Nguyen *et al.* (2006 ▶). For a related structure, see: Lai *et al.* (2007 ▶).
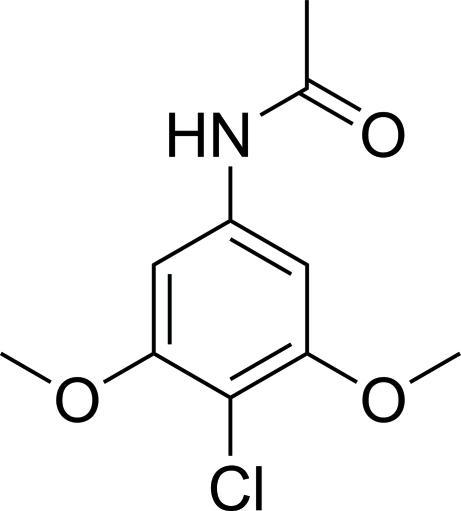


## Experimental

### 

#### Crystal data

C_10_H_12_ClNO_3_
*M* *_r_* = 229.66
Monoclinic, 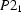

*a* = 11.1373 (14) Å
*b* = 15.1159 (19) Å
*c* = 14.2802 (18) Å
β = 111.9280 (10)°
*V* = 2230.1 (5) Å^3^
*Z* = 8
Mo *K*α radiation
μ = 0.33 mm^−1^
*T* = 296 K
0.30 × 0.20 × 0.20 mm

#### Data collection

Bruker SMART CCD area-detector diffractometer
Absorption correction: multi-scan (*SADABS*; Bruker, 2000 ▶) *T*_min_ = 0.908, *T*_max_ = 0.937
11396 measured reflections
6377 independent reflections
5985 reflections with *I* > 2σ(*I*)
*R*_int_ = 0.021

#### Refinement

*R*[*F* ^2^ > 2σ(*F* ^2^)] = 0.035
*wR*(*F* ^2^) = 0.105
*S* = 1.07
6377 reflections
570 parameters
38 restraints
H atoms treated by a mixture of independent and constrained refinement
Δρ_max_ = 0.25 e Å^−3^
Δρ_min_ = −0.18 e Å^−3^
Absolute structure: Flack (1983 ▶), 2293 Friedel pairs
Flack parameter: −0.02 (5)

### 

Data collection: *SMART* (Bruker, 2000 ▶); cell refinement: *SAINT* (Bruker, 2000 ▶); data reduction: *SAINT*; program(s) used to solve structure: *SHELXS97* (Sheldrick, 2008 ▶); program(s) used to refine structure: *SHELXL97* (Sheldrick, 2008 ▶); molecular graphics: *SHELXTL* (Sheldrick, 2008 ▶); software used to prepare material for publication: *SHELXTL*.

## Supplementary Material

Crystal structure: contains datablocks I, global. DOI: 10.1107/S1600536809013166/fl2238sup1.cif

Structure factors: contains datablocks I. DOI: 10.1107/S1600536809013166/fl2238Isup2.hkl

Additional supplementary materials:  crystallographic information; 3D view; checkCIF report

## Figures and Tables

**Table 1:** Hydrogen-bond geometry (Å, °)

*D*—H⋯*A*	*D*—H	H⋯*A*	*D*⋯*A*	*D*—H⋯*A*
N3—H3*A*⋯O11^i^	0.86 (3)	2.03 (4)	2.886 (4)	176 (3)
N2—H2*A*⋯O2^ii^	0.895 (18)	1.993 (19)	2.887 (4)	178 (3)
N1—H1*A*⋯O8^iii^	0.85 (4)	2.06 (4)	2.906 (4)	176 (4)
N4—H4*A*⋯O5	0.84 (4)	2.10 (4)	2.909 (4)	163 (3)

Symmetry codes: (i) 
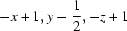
; (ii) 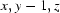; (iii) 
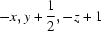
.

**Fig. 1.**
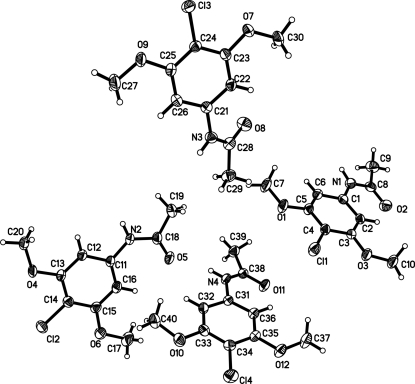
View of the molecule of (I) showing the atom-labelling scheme. Displacement ellipsoids are drawn at the 30% probability level and H atoms are shown as small spheres of arbitrary radii.

**Fig. 2.**
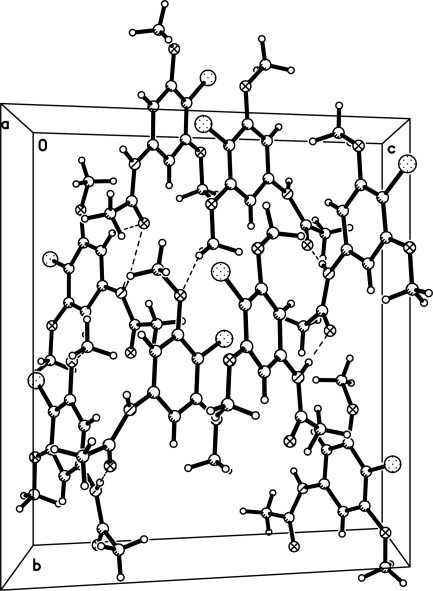
The crystal packing of (I), viewed along the a-axis. Hydrogen bonds are shown as dashed lines.

## supplementary crystallographic information

### Comment

The natural pyranoacridone acronycine was shown to exhibit a broad spectrum of activity against numerous experimental tumor models, including sarcoma, myeloma, carcinoma, and melanoma (Nguyen *et al.*, 2006). The title compound, (I, Fig. 1), can be used as an intermediate in the synthesis of acronycine derivatives. In this paper, we present the X-ray crystallographic analysis of (I), which crystallizes in the monoclinic space group P2(1) with four molecules in the asymmetric unit.

It is interesting to note that there are four independent molecules in the asymmetric unit which is different from a similar structure (Lai *et al.*, 2007) that has two molecules in the asymmetric unit. The dihedral angle between the acetamide groups and the benzene rings is 3.786 (3)°, 15.359 (4)°, 18.189 (3)° and 32.301 (6)° respectively. The torsion angles of the methoxy groups with respect to the benzene rings also vary (from 2.57 (1) to -15.43 (1)°. The molecules are linked into stacks by internolecular N-H···O hydrogen bonding (Fig. 2 and Table 1) with packing between the stacks stabilized by van der Waals forces.

### Experimental

To a solution of 3,5-dimethoxyanilide (195 mg, 1.0 mmol) in dry CH_2_Cl_2_ (4 ml) cooled with an ice-water bath was added *N*-chlorobutanimide (147 mg, 1.1 mmol) in portions. After  2 h, the reaction was warmed to room temperature and stirred for 30 min and  then filtered.  The resulting solution was evaporated to give a white solid which was further purified by column chromatography (CH_2_Cl_2_) to give (I) (yield 75%, m. p. 493 K). Single crystals of (I) were obtained by slow evaporation of a petroleum ether-ethyl acetate solution. After one week, single crystals suitable for X-ray diffraction were obtained respectively.

### Refinement

All the H atoms were placed in geometrically idealized positions and constrained to ride their parent atoms, with N—H = 0.840–0.895 Å, *U*_iso_(H) = 1.2*U*_eq_(N) and C—H = 0.93 Å, *U*_iso_(H) = 1.2*U*_eq_(C).

### Crystal data

**Table:**

C_10_H_12_ClNO_3_	*D*_x_ = 1.368 Mg m^−^^3^
*M**_r_* = 229.66	Melting point: 493 K
Monoclinic, *P*2_1_	Mo *K*α radiation, λ = 0.71073 Å
*a* = 11.1373 (14) Å	Cell parameters from 7547 reflections
*b* = 15.1159 (19) Å	θ = 2.4–27.2°
*c* = 14.2802 (18) Å	µ = 0.33 mm^−^^1^
β = 111.928 (1)°	*T* = 296 K
*V* = 2230.1 (5) Å^3^	Block, colourless
*Z* = 8	0.30 × 0.20 × 0.20 mm
*F*(000) = 960	

### Data collection

**Table:**

Bruker SMART CCD area-detector diffractometer	6377 independent reflections
Radiation source: fine-focus sealed tube	5985 reflections with *I* > 2σ(*I*)
graphite	*R*_int_ = 0.021
φ and ω scans	θ_max_ = 25.0°, θ_min_ = 2.0°
Absorption correction: multi-scan (*SADABS*; Bruker, 2000)	*h* = −12→13
*T*_min_ = 0.908, *T*_max_ = 0.937	*k* = −13→17
11396 measured reflections	*l* = −16→16

### Refinement

**Table:**

Refinement on *F*^2^	Hydrogen site location: inferred from neighbouring sites
Least-squares matrix: full	H atoms treated by a mixture of independent and constrained refinement
*R*[*F*^2^ > 2σ(*F*^2^)] = 0.035	*w* = 1/[σ^2^(*F*_o_^2^) + (0.0603*P*)^2^ + 0.5909*P*] where *P* = (*F*_o_^2^ + 2*F*_c_^2^)/3
*wR*(*F*^2^) = 0.105	(Δ/σ)_max_ = 0.001
*S* = 1.07	Δρ_max_ = 0.25 e Å^−^^3^
6377 reflections	Δρ_min_ = −0.17 e Å^−^^3^
570 parameters	Extinction correction: *SHELXL97* (Sheldrick, 2008), Fc^*^=kFc[1+0.001xFc^2^λ^3^/sin(2θ)]^-1/4^
38 restraints	Extinction coefficient: 0.0059 (8)
Primary atom site location: structure-invariant direct methods	Absolute structure: Flack (1983), 2293 Friedel pairs
Secondary atom site location: difference Fourier map	Flack parameter: −0.02 (5)

### Special details

**Table:**

Geometry. All e.s.d.'s (except the e.s.d. in the dihedral angle between two l.s. planes) are estimated using the full covariance matrix. The cell e.s.d.'s are taken into account individually in the estimation of e.s.d.'s in distances, angles and torsion angles; correlations between e.s.d.'s in cell parameters are only used when they are defined by crystal symmetry. An approximate (isotropic) treatment of cell e.s.d.'s is used for estimating e.s.d.'s involving l.s. planes.
Refinement. Refinement of *F*^2^ against ALL reflections. The weighted *R*-factor *wR* and goodness of fit *S* are based on *F*^2^, conventional *R*-factors *R* are based on *F*, with *F* set to zero for negative *F*^2^. The threshold expression of *F*^2^ > σ(*F*^2^) is used only for calculating *R*-factors(gt) *etc*. and is not relevant to the choice of reflections for refinement. *R*-factors based on *F*^2^ are statistically about twice as large as those based on *F*, and *R*- factors based on ALL data will be even larger.

### Fractional atomic coordinates and isotropic or equivalent isotropic displacement parameters (Å^2^)

**Table:**

	*x*	*y*	*z*	*U*_iso_*/*U*_eq_	
Cl1	0.50814 (9)	0.60141 (6)	0.49262 (8)	0.0642 (3)	
O1	0.2442 (2)	0.54965 (16)	0.3810 (2)	0.0611 (6)	
O2	0.1586 (3)	0.97634 (19)	0.3086 (2)	0.0662 (7)	
O3	0.5250 (2)	0.78822 (18)	0.4969 (2)	0.0568 (6)	
N1	0.0655 (3)	0.8428 (2)	0.3098 (2)	0.0503 (7)	
C1	0.1754 (3)	0.7879 (2)	0.3532 (2)	0.0421 (7)	
C2	0.2992 (3)	0.8216 (2)	0.4038 (2)	0.0424 (7)	
H2	0.3133	0.8824	0.4097	0.051*	
C3	0.4001 (3)	0.7634 (2)	0.4448 (2)	0.0421 (7)	
C4	0.3794 (3)	0.6724 (2)	0.4367 (2)	0.0440 (7)	
C5	0.2560 (3)	0.6400 (2)	0.3866 (2)	0.0455 (7)	
C6	0.1529 (3)	0.6973 (2)	0.3435 (2)	0.0462 (7)	
H6	0.0701	0.6755	0.3086	0.055*	
C7	0.1193 (4)	0.5133 (3)	0.3338 (4)	0.0762 (12)	
H7A	0.0653	0.5320	0.3691	0.114*	
H7B	0.1249	0.4499	0.3350	0.114*	
H7C	0.0826	0.5333	0.2651	0.114*	
C8	0.0608 (3)	0.9307 (3)	0.2914 (3)	0.0535 (8)	
C9	−0.0731 (4)	0.9684 (3)	0.2456 (4)	0.0721 (12)	
H9A	−0.0679	1.0316	0.2405	0.108*	
H9B	−0.1207	0.9541	0.2874	0.108*	
H9C	−0.1163	0.9438	0.1795	0.108*	
C10	0.5538 (4)	0.8806 (3)	0.5012 (3)	0.0665 (11)	
H10A	0.5279	0.9037	0.4339	0.100*	
H10B	0.6451	0.8893	0.5361	0.100*	
H10C	0.5077	0.9108	0.5366	0.100*	
Cl2	0.55954 (10)	−0.15609 (7)	0.01677 (8)	0.0650 (3)	
O4	0.3842 (2)	−0.20909 (15)	0.11098 (18)	0.0527 (6)	
O5	0.3629 (2)	0.21934 (17)	0.1938 (2)	0.0605 (6)	
O6	0.5970 (3)	0.03091 (19)	0.0383 (2)	0.0683 (7)	
N2	0.3041 (3)	0.07914 (18)	0.2169 (2)	0.0441 (6)	
C11	0.3702 (3)	0.0277 (2)	0.1693 (2)	0.0399 (7)	
C12	0.3470 (3)	−0.0642 (2)	0.1676 (2)	0.0420 (7)	
H12	0.2938	−0.0870	0.1986	0.050*	
C13	0.4040 (3)	−0.1200 (2)	0.1194 (2)	0.0415 (7)	
C14	0.4881 (3)	−0.0857 (2)	0.0775 (2)	0.0460 (7)	
C15	0.5123 (3)	0.0043 (2)	0.0808 (2)	0.0457 (7)	
C16	0.4517 (3)	0.0612 (2)	0.1258 (2)	0.0460 (7)	
H16	0.4662	0.1218	0.1264	0.055*	
C17	0.6186 (5)	0.1241 (3)	0.0333 (4)	0.0850 (15)	
H17A	0.5369	0.1539	0.0039	0.128*	
H17B	0.6689	0.1342	−0.0075	0.128*	
H17C	0.6643	0.1466	0.1001	0.128*	
C18	0.3005 (3)	0.1681 (2)	0.2249 (3)	0.0502 (8)	
C19	0.2165 (4)	0.2013 (3)	0.2789 (3)	0.0629 (10)	
H19A	0.2691	0.2130	0.3483	0.094*	
H19B	0.1529	0.1573	0.2757	0.094*	
H19C	0.1738	0.2547	0.2474	0.094*	
C20	0.2957 (4)	−0.2457 (3)	0.1515 (3)	0.0606 (10)	
H20A	0.3300	−0.2388	0.2236	0.091*	
H20B	0.2834	−0.3074	0.1349	0.091*	
H20C	0.2142	−0.2155	0.1233	0.091*	
Cl3	0.01359 (8)	−0.00462 (6)	1.02777 (6)	0.0524 (2)	
O7	0.0568 (2)	0.18003 (16)	1.00635 (18)	0.0531 (6)	
O8	0.1927 (2)	0.27104 (18)	0.73842 (19)	0.0609 (7)	
O9	0.1311 (3)	−0.10568 (15)	0.91356 (18)	0.0538 (6)	
N3	0.3026 (3)	0.1427 (2)	0.7915 (2)	0.0451 (6)	
C21	0.2327 (3)	0.1090 (2)	0.8477 (2)	0.0392 (7)	
C22	0.1802 (3)	0.1657 (2)	0.8994 (2)	0.0423 (7)	
H22	0.1904	0.2266	0.8972	0.051*	
C23	0.1126 (3)	0.1297 (2)	0.9542 (2)	0.0412 (7)	
C24	0.0979 (3)	0.0387 (2)	0.9583 (2)	0.0389 (6)	
C25	0.1523 (3)	−0.0168 (2)	0.9073 (2)	0.0405 (7)	
C26	0.2202 (3)	0.0183 (2)	0.8522 (2)	0.0428 (7)	
H26	0.2570	−0.0189	0.8185	0.051*	
C27	0.1790 (5)	−0.1637 (3)	0.8574 (3)	0.0682 (11)	
H27A	0.1397	−0.1494	0.7869	0.102*	
H27B	0.1584	−0.2237	0.8678	0.102*	
H27C	0.2712	−0.1572	0.8795	0.102*	
C28	0.2784 (3)	0.2203 (2)	0.7393 (2)	0.0454 (7)	
C29	0.3613 (3)	0.2385 (3)	0.6798 (3)	0.0573 (9)	
H29A	0.3143	0.2242	0.6102	0.086*	
H29B	0.4383	0.2031	0.7055	0.086*	
H29C	0.3846	0.3000	0.6856	0.086*	
C30	0.0697 (4)	0.2730 (2)	1.0017 (3)	0.0606 (9)	
H30A	0.1596	0.2887	1.0307	0.091*	
H30B	0.0240	0.3014	1.0386	0.091*	
H30C	0.0342	0.2919	0.9325	0.091*	
Cl4	1.03856 (8)	0.29948 (8)	0.49008 (8)	0.0684 (3)	
O10	0.8345 (2)	0.17496 (18)	0.3899 (2)	0.0619 (7)	
O11	0.4972 (2)	0.53597 (17)	0.23430 (19)	0.0573 (6)	
O12	0.9289 (2)	0.47290 (17)	0.47789 (18)	0.0566 (6)	
N4	0.4909 (2)	0.38854 (19)	0.2602 (2)	0.0429 (6)	
C31	0.6224 (3)	0.3698 (2)	0.3182 (2)	0.0361 (6)	
C32	0.6607 (3)	0.2822 (2)	0.3235 (2)	0.0430 (7)	
H32	0.6014	0.2386	0.2899	0.052*	
C33	0.7887 (3)	0.2602 (2)	0.3797 (2)	0.0457 (7)	
C34	0.8772 (3)	0.3252 (2)	0.4263 (2)	0.0437 (7)	
C35	0.8372 (3)	0.4127 (2)	0.4242 (2)	0.0410 (7)	
C36	0.7081 (3)	0.4349 (2)	0.3696 (2)	0.0403 (7)	
H36	0.6803	0.4931	0.3679	0.048*	
C37	0.8871 (4)	0.5614 (3)	0.4803 (3)	0.0678 (11)	
H37A	0.8620	0.5870	0.4142	0.102*	
H37B	0.9566	0.5953	0.5271	0.102*	
H37C	0.8146	0.5618	0.5012	0.102*	
C38	0.4380 (3)	0.4664 (2)	0.2209 (2)	0.0426 (7)	
C39	0.2963 (3)	0.4632 (3)	0.1571 (3)	0.0570 (9)	
H39A	0.2558	0.5171	0.1654	0.086*	
H39B	0.2572	0.4140	0.1774	0.086*	
H39C	0.2850	0.4565	0.0874	0.086*	
C40	0.7423 (4)	0.1055 (3)	0.3627 (3)	0.0641 (10)	
H40A	0.6810	0.1147	0.3945	0.096*	
H40B	0.7856	0.0500	0.3845	0.096*	
H40C	0.6977	0.1048	0.2907	0.096*	
H3A	0.361 (3)	0.109 (2)	0.786 (2)	0.039 (8)*	
H2A	0.261 (3)	0.047 (2)	0.247 (3)	0.055 (10)*	
H1A	−0.008 (4)	0.819 (3)	0.295 (3)	0.055 (11)*	
H4A	0.440 (3)	0.346 (3)	0.235 (3)	0.050 (10)*	

### Atomic displacement parameters (Å^2^)

**Table:**

	*U*^11^	*U*^22^	*U*^33^	*U*^12^	*U*^13^	*U*^23^
Cl1	0.0549 (5)	0.0394 (5)	0.0880 (6)	0.0025 (4)	0.0147 (4)	0.0066 (5)
O1	0.0557 (13)	0.0326 (14)	0.0833 (16)	−0.0071 (11)	0.0123 (12)	−0.0004 (12)
O2	0.0690 (16)	0.0501 (17)	0.0882 (18)	0.0024 (13)	0.0392 (14)	0.0134 (14)
O3	0.0495 (12)	0.0405 (15)	0.0749 (15)	−0.0088 (11)	0.0167 (11)	−0.0075 (12)
N1	0.0492 (16)	0.0429 (17)	0.0614 (17)	0.0034 (14)	0.0237 (14)	0.0054 (14)
C1	0.0497 (16)	0.0368 (19)	0.0453 (16)	0.0035 (14)	0.0241 (13)	0.0041 (14)
C2	0.0546 (17)	0.0311 (17)	0.0483 (16)	−0.0007 (14)	0.0271 (14)	−0.0018 (13)
C3	0.0442 (16)	0.0391 (19)	0.0463 (16)	−0.0040 (14)	0.0206 (14)	−0.0050 (14)
C4	0.0514 (17)	0.0325 (17)	0.0494 (17)	−0.0002 (14)	0.0204 (14)	−0.0004 (14)
C5	0.0557 (18)	0.0305 (17)	0.0524 (18)	−0.0046 (14)	0.0227 (15)	0.0050 (14)
C6	0.0453 (16)	0.041 (2)	0.0520 (17)	−0.0029 (15)	0.0184 (14)	−0.0011 (15)
C7	0.065 (2)	0.039 (2)	0.116 (3)	−0.0170 (19)	0.025 (2)	−0.006 (2)
C8	0.0609 (19)	0.049 (2)	0.062 (2)	0.0056 (18)	0.0365 (17)	0.0046 (18)
C9	0.071 (2)	0.058 (3)	0.095 (3)	0.019 (2)	0.041 (2)	0.018 (2)
C10	0.064 (2)	0.043 (2)	0.089 (3)	−0.0149 (18)	0.024 (2)	−0.008 (2)
Cl2	0.0837 (6)	0.0467 (5)	0.0848 (6)	0.0072 (5)	0.0548 (5)	0.0030 (5)
O4	0.0759 (15)	0.0264 (12)	0.0671 (14)	−0.0033 (11)	0.0398 (12)	−0.0016 (11)
O5	0.0622 (14)	0.0320 (14)	0.0868 (18)	−0.0095 (12)	0.0270 (13)	−0.0055 (13)
O6	0.0870 (18)	0.0430 (15)	0.0985 (19)	−0.0058 (14)	0.0619 (16)	0.0053 (14)
N2	0.0515 (14)	0.0282 (15)	0.0535 (15)	−0.0054 (12)	0.0206 (12)	−0.0033 (11)
C11	0.0403 (15)	0.0311 (17)	0.0436 (15)	−0.0012 (12)	0.0101 (12)	−0.0014 (13)
C12	0.0457 (15)	0.0331 (18)	0.0474 (16)	−0.0024 (14)	0.0175 (13)	0.0006 (14)
C13	0.0505 (16)	0.0300 (17)	0.0429 (15)	−0.0021 (13)	0.0160 (13)	0.0027 (13)
C14	0.0504 (16)	0.0415 (19)	0.0465 (16)	0.0045 (15)	0.0186 (14)	0.0042 (14)
C15	0.0467 (16)	0.0377 (18)	0.0533 (17)	−0.0019 (14)	0.0192 (14)	0.0093 (15)
C16	0.0492 (16)	0.0326 (18)	0.0502 (16)	−0.0017 (14)	0.0117 (14)	0.0044 (14)
C17	0.121 (4)	0.055 (3)	0.106 (3)	−0.027 (3)	0.074 (3)	0.002 (3)
C18	0.0464 (16)	0.0361 (19)	0.060 (2)	0.0015 (15)	0.0103 (15)	−0.0037 (16)
C19	0.074 (2)	0.040 (2)	0.078 (2)	0.0051 (18)	0.032 (2)	−0.0058 (19)
C20	0.086 (3)	0.039 (2)	0.073 (2)	−0.0149 (19)	0.048 (2)	−0.0115 (17)
Cl3	0.0530 (4)	0.0560 (6)	0.0536 (4)	−0.0101 (4)	0.0260 (4)	−0.0017 (4)
O7	0.0646 (14)	0.0405 (14)	0.0654 (14)	0.0032 (11)	0.0370 (12)	−0.0089 (11)
O8	0.0659 (15)	0.0546 (17)	0.0636 (15)	0.0218 (13)	0.0259 (12)	0.0196 (13)
O9	0.0757 (15)	0.0296 (13)	0.0619 (14)	−0.0054 (11)	0.0325 (12)	−0.0049 (10)
N3	0.0457 (14)	0.0422 (17)	0.0514 (15)	0.0054 (13)	0.0230 (12)	0.0058 (12)
C21	0.0375 (14)	0.0394 (18)	0.0391 (14)	0.0013 (13)	0.0123 (12)	0.0017 (14)
C22	0.0469 (16)	0.0300 (17)	0.0499 (17)	0.0006 (13)	0.0180 (14)	0.0005 (13)
C23	0.0390 (14)	0.0423 (19)	0.0407 (15)	0.0026 (13)	0.0132 (12)	−0.0074 (13)
C24	0.0391 (14)	0.0415 (18)	0.0357 (14)	−0.0034 (13)	0.0135 (12)	0.0009 (13)
C25	0.0463 (15)	0.0326 (17)	0.0396 (14)	−0.0005 (13)	0.0125 (12)	−0.0005 (13)
C26	0.0477 (16)	0.0401 (19)	0.0419 (15)	0.0062 (14)	0.0181 (13)	−0.0024 (13)
C27	0.102 (3)	0.035 (2)	0.078 (3)	0.005 (2)	0.046 (2)	−0.0047 (19)
C28	0.0445 (15)	0.044 (2)	0.0424 (15)	−0.0005 (15)	0.0109 (13)	0.0064 (15)
C29	0.0567 (19)	0.056 (2)	0.061 (2)	−0.0024 (18)	0.0250 (17)	0.0147 (18)
C30	0.077 (2)	0.036 (2)	0.075 (2)	0.0081 (17)	0.036 (2)	−0.0091 (18)
Cl4	0.0425 (4)	0.0693 (7)	0.0779 (6)	0.0103 (4)	0.0048 (4)	0.0099 (5)
O10	0.0555 (14)	0.0418 (15)	0.0805 (17)	0.0127 (12)	0.0163 (12)	0.0019 (13)
O11	0.0465 (12)	0.0368 (13)	0.0775 (16)	−0.0083 (11)	0.0106 (11)	0.0043 (12)
O12	0.0441 (12)	0.0495 (16)	0.0607 (13)	−0.0096 (11)	0.0018 (10)	0.0048 (11)
N4	0.0352 (12)	0.0349 (16)	0.0527 (15)	−0.0069 (12)	0.0097 (11)	−0.0019 (12)
C31	0.0365 (13)	0.0358 (18)	0.0352 (13)	−0.0004 (12)	0.0124 (11)	0.0028 (12)
C32	0.0430 (15)	0.0372 (18)	0.0457 (16)	0.0001 (13)	0.0128 (13)	−0.0035 (14)
C33	0.0473 (16)	0.043 (2)	0.0460 (16)	0.0074 (15)	0.0168 (14)	0.0003 (14)
C34	0.0389 (15)	0.048 (2)	0.0422 (15)	0.0054 (14)	0.0124 (12)	0.0085 (14)
C35	0.0404 (15)	0.0426 (19)	0.0367 (14)	−0.0075 (14)	0.0106 (12)	0.0024 (13)
C36	0.0402 (14)	0.0378 (17)	0.0393 (14)	−0.0004 (14)	0.0106 (12)	0.0056 (13)
C37	0.060 (2)	0.047 (2)	0.073 (2)	−0.0112 (18)	−0.0012 (18)	−0.0011 (19)
C38	0.0385 (15)	0.0393 (19)	0.0488 (16)	−0.0008 (14)	0.0149 (13)	−0.0010 (14)
C39	0.0402 (16)	0.047 (2)	0.075 (2)	−0.0010 (15)	0.0111 (16)	−0.0036 (18)
C40	0.074 (2)	0.045 (2)	0.070 (2)	0.008 (2)	0.0227 (19)	−0.0044 (19)

### Geometric parameters (Å, °)

**Table:**

Cl1—C4	1.729 (3)	Cl3—C24	1.729 (3)
O1—C5	1.371 (4)	O7—C23	1.366 (4)
O1—C7	1.412 (5)	O7—C30	1.417 (4)
O2—C8	1.234 (4)	O8—C28	1.220 (4)
O3—C3	1.363 (4)	O9—C25	1.372 (4)
O3—C10	1.428 (5)	O9—C27	1.420 (4)
N1—C8	1.351 (5)	N3—C28	1.361 (4)
N1—C1	1.416 (4)	N3—C21	1.405 (4)
N1—H1A	0.85 (4)	N3—H3A	0.86 (3)
C1—C6	1.390 (5)	C21—C26	1.383 (5)
C1—C2	1.392 (4)	C21—C22	1.394 (4)
C2—C3	1.375 (4)	C22—C23	1.384 (4)
C2—H2	0.9300	C22—H22	0.9300
C3—C4	1.393 (5)	C23—C24	1.389 (5)
C4—C5	1.381 (5)	C24—C25	1.390 (4)
C5—C6	1.385 (5)	C25—C26	1.384 (4)
C6—H6	0.9300	C26—H26	0.9300
C7—H7A	0.9600	C27—H27A	0.9600
C7—H7B	0.9600	C27—H27B	0.9600
C7—H7C	0.9600	C27—H27C	0.9600
C8—C9	1.500 (5)	C28—C29	1.495 (5)
C9—H9A	0.9600	C29—H29A	0.9600
C9—H9B	0.9600	C29—H29B	0.9600
C9—H9C	0.9600	C29—H29C	0.9600
C10—H10A	0.9600	C30—H30A	0.9600
C10—H10B	0.9600	C30—H30B	0.9600
C10—H10C	0.9600	C30—H30C	0.9600
Cl2—C14	1.741 (3)	Cl4—C34	1.729 (3)
O4—C13	1.362 (4)	O10—C33	1.373 (4)
O4—C20	1.428 (4)	O10—C40	1.418 (5)
O5—C18	1.229 (4)	O11—C38	1.219 (4)
O6—C15	1.359 (4)	O12—C35	1.369 (4)
O6—C17	1.436 (5)	O12—C37	1.421 (5)
N2—C18	1.351 (4)	N4—C38	1.341 (4)
N2—C11	1.408 (4)	N4—C31	1.416 (4)
N2—H2A	0.895 (18)	N4—H4A	0.84 (4)
C11—C16	1.374 (5)	C31—C36	1.376 (4)
C11—C12	1.411 (5)	C31—C32	1.385 (5)
C12—C13	1.384 (5)	C32—C33	1.390 (4)
C12—H12	0.9300	C32—H32	0.9300
C13—C14	1.388 (4)	C33—C34	1.375 (5)
C14—C15	1.385 (5)	C34—C35	1.392 (5)
C15—C16	1.390 (5)	C35—C36	1.397 (4)
C16—H16	0.9300	C36—H36	0.9300
C17—H17A	0.9600	C37—H37A	0.9600
C17—H17B	0.9600	C37—H37B	0.9600
C17—H17C	0.9600	C37—H37C	0.9600
C18—C19	1.504 (5)	C38—C39	1.500 (4)
C19—H19A	0.9600	C39—H39A	0.9600
C19—H19B	0.9600	C39—H39B	0.9600
C19—H19C	0.9600	C39—H39C	0.9600
C20—H20A	0.9600	C40—H40A	0.9600
C20—H20B	0.9600	C40—H40B	0.9600
C20—H20C	0.9600	C40—H40C	0.9600
			
C5—O1—C7	118.1 (3)	C23—O7—C30	116.9 (3)
C3—O3—C10	117.4 (3)	C25—O9—C27	117.2 (3)
C8—N1—C1	128.7 (3)	C28—N3—C21	125.9 (3)
C8—N1—H1A	113 (3)	C28—N3—H3A	117 (2)
C1—N1—H1A	118 (3)	C21—N3—H3A	116 (2)
C6—C1—C2	121.3 (3)	C26—C21—C22	120.9 (3)
C6—C1—N1	116.1 (3)	C26—C21—N3	118.3 (3)
C2—C1—N1	122.6 (3)	C22—C21—N3	120.7 (3)
C3—C2—C1	118.7 (3)	C23—C22—C21	118.9 (3)
C3—C2—H2	120.7	C23—C22—H22	120.6
C1—C2—H2	120.7	C21—C22—H22	120.6
O3—C3—C2	124.2 (3)	O7—C23—C22	122.9 (3)
O3—C3—C4	115.0 (3)	O7—C23—C24	116.4 (3)
C2—C3—C4	120.9 (3)	C22—C23—C24	120.7 (3)
C5—C4—C3	119.7 (3)	C23—C24—C25	119.6 (3)
C5—C4—Cl1	120.9 (3)	C23—C24—Cl3	119.8 (2)
C3—C4—Cl1	119.4 (2)	C25—C24—Cl3	120.6 (3)
O1—C5—C4	116.0 (3)	O9—C25—C26	123.8 (3)
O1—C5—C6	123.5 (3)	O9—C25—C24	115.9 (3)
C4—C5—C6	120.5 (3)	C26—C25—C24	120.2 (3)
C5—C6—C1	118.9 (3)	C21—C26—C25	119.6 (3)
C5—C6—H6	120.5	C21—C26—H26	120.2
C1—C6—H6	120.5	C25—C26—H26	120.2
O1—C7—H7A	109.5	O9—C27—H27A	109.5
O1—C7—H7B	109.5	O9—C27—H27B	109.5
H7A—C7—H7B	109.5	H27A—C27—H27B	109.5
O1—C7—H7C	109.5	O9—C27—H27C	109.5
H7A—C7—H7C	109.5	H27A—C27—H27C	109.5
H7B—C7—H7C	109.5	H27B—C27—H27C	109.5
O2—C8—N1	122.9 (3)	O8—C28—N3	122.8 (3)
O2—C8—C9	122.3 (4)	O8—C28—C29	121.6 (3)
N1—C8—C9	114.8 (3)	N3—C28—C29	115.6 (3)
C8—C9—H9A	109.5	C28—C29—H29A	109.5
C8—C9—H9B	109.5	C28—C29—H29B	109.5
H9A—C9—H9B	109.5	H29A—C29—H29B	109.5
C8—C9—H9C	109.5	C28—C29—H29C	109.5
H9A—C9—H9C	109.5	H29A—C29—H29C	109.5
H9B—C9—H9C	109.5	H29B—C29—H29C	109.5
O3—C10—H10A	109.5	O7—C30—H30A	109.5
O3—C10—H10B	109.5	O7—C30—H30B	109.5
H10A—C10—H10B	109.5	H30A—C30—H30B	109.5
O3—C10—H10C	109.5	O7—C30—H30C	109.5
H10A—C10—H10C	109.5	H30A—C30—H30C	109.5
H10B—C10—H10C	109.5	H30B—C30—H30C	109.5
C13—O4—C20	117.4 (3)	C33—O10—C40	117.6 (3)
C15—O6—C17	118.0 (3)	C35—O12—C37	117.2 (3)
C18—N2—C11	128.9 (3)	C38—N4—C31	128.1 (3)
C18—N2—H2A	118 (2)	C38—N4—H4A	112 (3)
C11—N2—H2A	113 (2)	C31—N4—H4A	119 (3)
C16—C11—N2	124.6 (3)	C36—C31—C32	121.1 (3)
C16—C11—C12	120.1 (3)	C36—C31—N4	121.9 (3)
N2—C11—C12	115.3 (3)	C32—C31—N4	117.0 (3)
C13—C12—C11	119.7 (3)	C31—C32—C33	119.3 (3)
C13—C12—H12	120.2	C31—C32—H32	120.4
C11—C12—H12	120.2	C33—C32—H32	120.4
O4—C13—C12	123.9 (3)	O10—C33—C34	116.6 (3)
O4—C13—C14	116.4 (3)	O10—C33—C32	123.1 (3)
C12—C13—C14	119.7 (3)	C34—C33—C32	120.2 (3)
C15—C14—C13	120.4 (3)	C33—C34—C35	120.2 (3)
C15—C14—Cl2	119.9 (2)	C33—C34—Cl4	120.7 (3)
C13—C14—Cl2	119.6 (3)	C35—C34—Cl4	119.1 (2)
O6—C15—C14	115.7 (3)	O12—C35—C34	117.0 (3)
O6—C15—C16	124.1 (3)	O12—C35—C36	123.4 (3)
C14—C15—C16	120.1 (3)	C34—C35—C36	119.7 (3)
C11—C16—C15	119.9 (3)	C31—C36—C35	119.3 (3)
C11—C16—H16	120.1	C31—C36—H36	120.3
C15—C16—H16	120.1	C35—C36—H36	120.3
O6—C17—H17A	109.5	O12—C37—H37A	109.5
O6—C17—H17B	109.5	O12—C37—H37B	109.5
H17A—C17—H17B	109.5	H37A—C37—H37B	109.5
O6—C17—H17C	109.5	O12—C37—H37C	109.5
H17A—C17—H17C	109.5	H37A—C37—H37C	109.5
H17B—C17—H17C	109.5	H37B—C37—H37C	109.5
O5—C18—N2	123.9 (3)	O11—C38—N4	124.3 (3)
O5—C18—C19	121.3 (3)	O11—C38—C39	120.5 (3)
N2—C18—C19	114.8 (3)	N4—C38—C39	115.1 (3)
C18—C19—H19A	109.5	C38—C39—H39A	109.5
C18—C19—H19B	109.5	C38—C39—H39B	109.5
H19A—C19—H19B	109.5	H39A—C39—H39B	109.5
C18—C19—H19C	109.5	C38—C39—H39C	109.5
H19A—C19—H19C	109.5	H39A—C39—H39C	109.5
H19B—C19—H19C	109.5	H39B—C39—H39C	109.5
O4—C20—H20A	109.5	O10—C40—H40A	109.5
O4—C20—H20B	109.5	O10—C40—H40B	109.5
H20A—C20—H20B	109.5	H40A—C40—H40B	109.5
O4—C20—H20C	109.5	O10—C40—H40C	109.5
H20A—C20—H20C	109.5	H40A—C40—H40C	109.5
H20B—C20—H20C	109.5	H40B—C40—H40C	109.5

### Hydrogen-bond geometry (Å, °)

**Table:**

*D*—H···*A*	*D*—H	H···*A*	*D*···*A*	*D*—H···*A*
N3—H3A···O11^i^	0.86 (3)	2.03 (4)	2.886 (4)	176 (3)
N2—H2A···O2^ii^	0.90 (2)	1.99 (2)	2.887 (4)	178 (3)
N1—H1A···O8^iii^	0.85 (4)	2.06 (4)	2.906 (4)	176 (4)
N4—H4A···O5	0.84 (4)	2.10 (4)	2.909 (4)	163 (3)

Symmetry codes: (i) −*x*+1, *y*−1/2, −*z*+1; (ii) *x*, *y*−1, *z*; (iii) −*x*, *y*+1/2, −*z*+1.
